# Blood transfusion requirements and filter longevity with regional citrate anticoagulation compared with heparin

**DOI:** 10.1186/2197-425X-3-S1-A462

**Published:** 2015-10-01

**Authors:** E Burford, E Walter, A Beverly

**Affiliations:** Royal Surrey County Hospital, Intensive Care Unit, Guildford, United Kingdom

## Introduction

Citrate is the recommended anticoagulant for continuous renal replacement therapy (RRT) [1], and is thought to confer numerous benefits, including more continuous hours of filtration, fewer total circuits used, less overall cost and maybe improved patient and kidney survival when compared with heparin anticoagulation [2]. Our ICU changed from heparin to citrate anticoagulation in June 2014. Our unit uses a transfusion trigger of 7g/dl [3] unless the clinical situation dictates otherwise.

## Objectives

This observational study is to investigate blood transfusion requirements and haemofilter set life before and after the change from systemic heparin to citrate regional anticoagulation, to add to growing international evidence, and to inform local practice.

## Methods

Data were collected on set life and the number of blood transfusions patients received during the period of filtration, and in the 24 hours afterwards. Data on patients anticoagulated with heparin were collected retrospectively up until the day of changing to citrate. Data during citrate anticoagulation were collected prospectively, from the day of changing, onwards.

## Results

6 months of filtration with heparin and 3 months of citrate were observed. 97 patients required RRT (65 with heparin, 28 with citrate, 4 with both), for a total of 556 days. Table one shows the comparison between heparin and citrate. There was no difference in the average duration of filtration required between the 2 systems. Filter life increased from 0.86 days with heparin, to 1.55 days with citrate (p = 0.007 by Student t-test). The number of transfusions required fell from 0.33 units per day with heparin, to 0.25 units per day with citrate (p > 0.05).

## Conclusions

Regional citrate anticoagulation increases filter life when compared with systemic heparinisation, with significant cost and other savings. There is a non-significant trend towards a reduction in blood transfusion requirements.

**Table 1:** Comparison of citrate with heparin anticoagulation.

	Heparin	Citrate
Renal days	357	199
Median renal days per patient episode	3	3
Number of filters used	408	128
Total filter cost (•)	32807.28	10292.48
Renal days per filter	0.86	1.55
Filter cost per renal day (•)	91.89	51.72
Blood transfusions per day	0.33	0.25

## References

[1] Kidney Disease: Improving Global Outcomes (KDIGO) Acute Kidney Injury Work Group. Kidney inter., Suppl. 2012, 2: 1-138. KDIGO Clinical Practice Guideline for Acute Kidney Injury

[2] Oudemans-van Straaten HM, Kellum JA, Bellomo R (2010). Clinical review: Anticoagulation for continuous renal replacement therapy - heparin or citrate?. Crit Care.

[3] Hébert PC, Wells G, Blajchman MA, Marshall J, Martin C, Pagliarello G (1999). A Multicenter, Randomized, Controlled Clinical Trial of Transfusion Requirements in Critical Care. N Engl J Med.

